# Nisin ZP, a Bacteriocin and Food Preservative, Inhibits Head and Neck Cancer Tumorigenesis and Prolongs Survival

**DOI:** 10.1371/journal.pone.0131008

**Published:** 2015-07-01

**Authors:** Pachiyappan Kamarajan, Takayuki Hayami, Bibiana Matte, Yang Liu, Theodora Danciu, Ayyalusamy Ramamoorthy, Francis Worden, Sunil Kapila, Yvonne Kapila

**Affiliations:** 1 Department of Periodontics and Oral Medicine, School of Dentistry, University of Michigan, Ann Arbor, Michigan, United States of America; 2 Department of Orthodontics and Pediatric Dentistry, School of Dentistry, University of Michigan, Ann Arbor, Michigan, United States of America; 3 Department of Chemistry and Biophysics, College of Literature, Science, and the Arts, University of Michigan, Ann Arbor, Michigan, United States of America; 4 Department of Internal Medicine, School of Medicine, University of Michigan, Ann Arbor, Michigan, United States of America; Louisiana State University Health Sciences Center, UNITED STATES

## Abstract

The use of small antimicrobial peptides or bacteriocins, like nisin, to treat cancer is a new approach that holds great promise. Nisin exemplifies this new approach because it has been used safely in humans for many years as a food preservative, and recent laboratory studies support its anti-tumor potential in head and neck cancer. Previously, we showed that nisin (2.5%, low content) has antitumor potential in head and neck squamous cell carcinoma (HNSCC) *in vitro* and *in vivo*. The current studies explored a naturally occurring variant of nisin (nisin ZP; 95%, high content) for its antitumor effects *in vitro* and *in vivo*. Nisin ZP induced the greatest level of apoptosis in HNSCC cells compared to low content nisin. HNSCC cells treated with increasing concentrations of nisin ZP exhibited increasing levels of apoptosis and decreasing levels of cell proliferation, clonogenic capacity, and sphere formation. Nisin ZP induced apoptosis through a calpain-dependent pathway in HNSCC cells but not in human oral keratinocytes. Nisin ZP also induced apoptosis dose-dependently in human umbilical vein endothelial cells (HUVEC) with concomitant decreases in vascular sprout formation *in vitro* and reduced intratumoral microvessel density *in vivo*. Nisin ZP reduced tumorigenesis *in vivo* and long-term treatment with nisin ZP extended survival. In addition, nisin treated mice exhibited normal organ histology with no evidence of inflammation, fibrosis or necrosis. In summary, nisin ZP exhibits greater antitumor effects than low content nisin, and thus has the potential to serve as a novel therapeutic for HNSCC.

## Introduction

Head and neck squamous cell carcinoma (HNSCC) is the sixth leading cause of death worldwide. Patients diagnosed with HNSCC are treated with surgery, radiotherapy and chemotherapy. Given its anatomical location, HNSCC surgical resection is often destructive and, frequently, complete removal of the tumor mass is not a viable option, often rendering these patients with incurable disease following treatments with chemoradiotherapy [1]. There are limited chemotherapeutic options for patients once their disease is no longer amenable to cure, and the low 5-year survival rates for patients with metastatic HNSCC have not improved in decades [2,3]. These facts emphasize the urgency of improving treatment options for such patients.

A few reports have suggested that antimicrobial peptides or bacteriocins have cytotoxic effects against cancer cells [4–8]. Given this interesting premise, we explored the cytotoxic and antitumor properties of the antimicrobial peptide, nisin, and found that it blocks HNSCC tumorigenesis [9]. Nisin is a 34-amino acid polycyclic antibacterial peptide that is produced by fermentation of the gram-positive bacterium *Lactococcus lactis*. Many antibacterial agents are effective against similar bacterial species; however, nisin has broad-spectrum effects, as it also inhibits gram-negative bacteria [10]. Nisin is not toxic to animals and is safe for human consumption [11]. Nisin was approved for human use as a food preservative by the world health organization (WHO) in 1969 and by the food and drug administration (FDA) in 1988, and it has been given a generally-regarded-as-safe (GRAS) designation by the FDA [12]. It is estimated that 0.94 to 2.24 mg of nisin are consumed per person per day in the US [12]. While bacteriocins, like nisin, have been used for decades in preventing bacterial growth in foods, they have only recently been tested for prevention of growth or induction of apoptosis of cancer cells. Apoptosis or programmed cell death is a natural process that eliminates unwanted or older cells. However, cancer cells are resistant to apoptosis. Thus, there is great effort to understand the mechanisms that regulate apoptosis in these cells so that novel agents can be developed to induce apoptosis of cancer cells. Nisin may serve in this capacity and development of nisin as a cancer therapeutic can be readily pursued following dosing determinations.

Recently, we reported that nisin, may serve as a novel potential therapeutic for treating HNSCC [9]. Nisin mediates these effects by inducing preferential apoptosis, cell cycle arrest, and reducing cell proliferation in HNSCC cells, compared to primary keratinocytes. Nisin also reduces HNSCC tumorigenesis in vivo. Mechanistically, nisin exerts these effects on HNSCC, in part, through cation transport regulator homolog 1 (CHAC1), a proapoptotic cation transport regulator and through a concomitant CHAC1-independent influx of extracellular calcium [9,13]. These findings support the use of nisin as a potentially novel therapeutic for HNSCC, and since nisin is safe for human consumption as a food preservative, its translation into a clinical setting may be facilitated.

Nisin acts by altering the integrity of the cellular membrane and forming short-lived pores, thereby changing the membrane potential [14]. Nisin becomes immersed in the cell membrane through the cationic portions of the amino acids extending to one side of the molecule. These cationic portions interact with the negatively charged phospholipid heads, while the hydrophobic portion of nisin interacts with the membrane core [15]. Nisin's involvement with the membrane, therefore, mediates phospholipid reorganization and allows an influx of ions [14,16]. Since HNSCC cells and primary keratinocytes differ in their lipid membrane composition and function and response to calcium fluxes, nisin's ability to alter the transmembrane potential and membrane composition of cells may lead to differential effects on these cells [17–22]. Indeed, our data support this premise as the basis for the nisin-mediated differential apoptotic cell death and reduced proliferation of HNSCC cells compared to primary keratinocytes [9].

Given nisin's role as a safe bacteriocin for food preservation, and the knowledge that other bacteriocins possess proapoptotic properties against cancer cells, our findings on the effects of nisin on HNSCC tumorigenesis help provide a basis for nisin as a potential novel therapeutic for HNSCC. Thus, our focus in the current study was to extend this baseline knowledge. Previously, we used a 2.5% nisin (low content) formulation. In this study, our aim was to test the efficacy of a 95% nisin (high content) on HNSCC cell apoptosis and proliferation in vitro and on tumorigenesis in vivo. Specifically, we focused on its translational potential by examining a highly pure, food grade form of nisin for its in vivo and long term effects. To this end, we tested two naturally occurring, high nisin content variants, nisin ZP and nisin AP. Considering nisin’s safety for human consumption and given its in vitro and in vivo efficacy in HNSCC, nisin could be developed as a novel cancer therapeutic for HNSCC.

## Materials and Methods

### Institutional Review Board Approval

This study has been approved by and was conducted in accordance with the regulations set for by the institutional review board and the committee on the use and care of animals at the University of Michigan.

### Cell culture

Four human HNSCC cell lines were used for these studies. HNSCC cell line authentication and origin was provided by their sources and published extensively. The human HNSCC cell lines, UM-SCC-17B (supraglottis/soft tissue-neck) and UM-SCC-14A (floor of mouth) were provided by Dr. Thomas Carey (Professor, University of Michigan, MI) [23]. The oral SCC cell line HSC-3 (tongue) was provided by Dr. Randall Kramer (Professor, University of California, San Francisco, CA) [24]. The oral SCC cell line, OSCC-3 (tongue) was provided by Dr. Mark Lingen (Professor, University of Chicago). HNSCC cells were maintained in Dulbecco’s modified Eagle’s medium (DMEM) containing 10% fetal bovine serum, 1% penicillin, and 1% streptomycin. Normal-human umbilical vein endothelial cells (HUVECs), and accompanying cell culture media and supplements (EGM-2 Bullet Kits) were obtained from Lonza (Allendale, NJ). Human recombinant VEGF165 was obtained from Sigma-Aldrich (St. Louis, MO) and Matrigel Matrix was obtained from BD Biosciences (San Jose, CA). HUVECs were cultured in EGM-2 basal media at 37°C in 5% CO_2_.

### Nisin

Nisin A and nisin Z are two naturally occurring variants of nisin that are produced by fermentation of *Lactococcus* lactis and they differ by a single amino acid residue at position 27; histidine in nisin A and asparagine in nisin Z [25]. High content forms of these two variants, nisin ZP and nisin AP (95% content/ultrapure; % weight/weight; hydrous potency ≥38,000 IU/mg) were purchased from Handary (Brussels, Belgium) and low content nisin A (2.5% content in balance sodium chloride and denatured milk solids; potency ≥1,000,000 per IU/g) was purchased from Sigma-Aldrich (N5764). Nisin ZP and AP and low content nisin were reconstituted in water and used for all experiments.

### Cell Proliferation and Colony Formation Assays

To determine the effect of nisin on cell proliferation, the CyQUANT NF Cell Proliferation Assay Kit was used according to manufacturer’s instructions (Invitrogen/Life Technologies, Grand Island, NY). For colony formation assays, 2000 cells were seeded in a 6-well plate. Following overnight incubation, the cells were treated with different concentrations of nisin ZP for 48 hours. After treatment, fresh media was added every 2 days for a period of 10 days. Colonies were then stained with 0.5% crystal violet and colonies that contained >50 cells were counted. Experiments were repeated in triplicate.

### Orasphere Assay

Sphere assays were used to assess anchorage-independent growth, a property thought to contribute to metastatic potential. HNSCC oraspheres (UM-SCC-17B) were prepared as previously reported [26–29]. In brief, cells that survive anchorage withdrawal form multicellular aggregates or oraspheres. Oraspheres were developed by maintaining cells under suspension conditions on poly (2-hydroxyethyl methacrylate) (poly-HEMA) coated plates (7.5 mg/mL in 95% ethanol, Sigma-Aldrich) for 36 hours. An orasphere is defined as an aggregate of cells that is at least 50 μm in diameter. The total area occupied by oraspheres in each well was quantified for each treatment condition using NIS-Elements BR4.13.04 imaging software. Experiments were performed in triplicate. Nisin was added to each well at the time of cell plating.

### Apoptosis Assays

To determine the effects of three different forms of nisin on HNSCC cell apoptosis three different assays were performed. Apoptosis was assessed in nisin treated cells using a direct staining method, a flow cytometry-based assay, and a western blotting approach to evaluate known apoptotic signaling proteins.

### Apoptosis: Staining and Microscopy

Ethidium bromide and acridine orange (EB/AO) staining was used to measure apoptosis as previously described [30]. For these assays, cells were plated in 96-well plates at 2 × 10^4^ cells/cm^2^, and after 24 hours, treated with various concentration of nisin (0, 100, 200, 400 and 800 μg/mL) for 24 hours. Cells were then stained with an EB/AO stain. EB was obtained from Bio-rad (Berkeley, CA) and AO was obtained from Acros Organics (Geel, Belgium). The EB/AO dye reagent was comprised of 100μg/ml of ethidium bromide and 100μg/ml of acridine orange in PBS. The EB/AO dye was added to each well, removed, and then images of stained cells were captured using a microscope equipped with a digital imaging system (Eclipse 50i Nikon, Melville, NY). Cells were counted and, based on their color, were classified as either vital, apoptotic or necrotic. AO permeates all cells and makes the nuclei appear green. EB is only taken up by cells when cytoplasmic membrane integrity is lost, and it stains the nuclei so they appear red. Thus, early apoptotic cells appear green with bright green dots in the nuclei due to chromatin condensation and nuclear fragmentation. Late apoptotic cells appear orange (combination of green and red colors) with more significant chromatin condensation and nuclear fragmentation. Necrotic cells appear orange but their nuclear morphology resembles that of viable cells, thus there is an absence of chromatin condensation. The percentage of cells in each category was determined by counting a minimum of 100 cells. Experiments were performed in triplicate.

### Apoptosis: Flow cytometry

The percentage of apoptotic cells induced by nisin treatment was determined by flow cytometry. Briefly, cells were detached by incubation with enzyme-free dissociation buffer (Invitrogen), pelleted by centrifugation, and stained with Annexin V (BD Pharmingen) for analysis by flow cytometry (FACSDiVa Cell sorter, Becton Dickinson).

### Apoptosis: Western Blotting

To evaluate the effects of nisin ZP on the expression of pro-apoptotic proteins in HNSCC cells, we performed Western blot analyses of UM-SCC-17B cells that were treated with nisin ZP for 18 hours. A calpain inhibitor was also examined in this context for its ability to reverse the apoptotic effects induced by nisin ZP; namely blocking PARP cleavage. The calpain inhibitor was purchased from Sigma-Aldrich (A6185, St. Louis, MO), reconstituted in water, and used at a concentration of 500 nM. Following various treatments, cells were collected, washed once with phosphate-buffered saline and lysed for 30 minutes while on ice in radioimmunoprecipitatation assay (RIPA) buffer (R0278, Sigma-Aldrich) that contained a 1% protease inhibitor cocktail (P8340, Sigma-Aldrich). Lysates were adjusted for protein concentration with the BCA protein assay kit (Bio-Rad, Berkeley, CA). Protein lysates were resolved by sodium dodecyl sulfate-polyacrylamide gel electrophoresis (SDS-PAGE) and transferred to Immobilon-P membranes (Millipore, Billerica, MA). Western blot analyses were performed using an anti-poly (ADP-ribose) polymerase-1 (PARP, SC-7150, Santa Cruz Biotechnology, Santa Cruz, CA) primary antibody, a caspase-3 primary antibody (SC-7148, Santa Cruz Biotechnology), a calpain primary antibody (MAB3083, Millipore) followed by a horseradish peroxidase-conjugated anti-rabbit antibody (SC-2004, Santa Cruz Biotechnology) or anti-mouse antibody (A9044, Sigma-Aldrich). Blots were then developed with the ECL-plus detection system (Pierce). To evaluate the samples for equal protein loading, membranes were stripped and reprobed with an anti-β-actin antibody (SC-1615, Santa Cruz Biotechnology).

### In Vitro Angiogenesis Assay

To determine the effect of nisin ZP on angiogenesis, we performed in vitro sprout assays [31]. For sprout assays, HUVECs were trypsinized and suspended in endothelial cell growth media (EGM-2) containing 50 ng/ml of recombinant vascular endothelial growth factor (VEGF) and various concentrations of nisin ZP (0, 100, 400 and 800 μg/mL). Cells were seeded at 1 × 10^4^ cells/cm^2^ in Matrigel coated 96-well plates and incubated overnight. Images of the sprouts were captured using the EVOS XL Core Imaging System (Life Technologies, Grand Island, NY), then total sprout length was measured using Image J (National Institutes of Health). Assays were performed in triplicate.

### Immunohistochemical Analyses

To evaluate nisin’s effects on angiogenesis in vivo, immunohistochemical analyses were used to evaluate CD31 expression, an endothelial cell adhesion molecule, in mouse tumor tissue sections. Briefly, after antigen retrieval by microwave pretreatment (citrate buffer, 10 mM, pH 6.0), slides were incubated with a CD31 primary antibody (DIA-310, Dianova, Hamburg, Germany) overnight at 4°C. After diaminobenzidine chromogen (DAB) reaction, slides were counterstained with routine hematoxylin. Immunohistochemical staining for CD31 was graded and scored by a pathologist in a blinded manner.

### Oral cancer mouse model

To examine the in vivo antitumor effects of nisin ZP, an oral cancer floor-of-mouth mouse model was used. UM-SCC-17B cells were injected submucosally into the floor of the mouth in mice as previously described [9, 28, 29, 32]. All protocols for the in vivo studies were approved by the Committee on the Use and Care of Animals at the University of Michigan. Specifically, cells were grown to 70% confluence, suspended in DMEM, mixed with an equal volume of growth factor-reduced Matrigel basement membrane matrix (BD Biosciences, San Jose, CA) at a final concentration of 1.25 x 10^4^/0.05 mL. Six-week-old athymic nude mice (NCr-nu/nu strain, NCI, Frederick, MD) were anesthetized by intraperitoneal injection with 100 mg/kg ketamine and 10 mg/kg xylazine. A total volume of 0.05 mL of SCC cell/Matrigel suspension was injected submucosally into the floor of the mouth. Three weeks after tumor cell injections and upon confirmation that tumors were established, animals were equally distributed into six groups: a control group that was given water (equal volume/control) by oral gavage for 3 weeks and five different treatment groups that were given nisin treatment by oral gavage for 3 weeks. Following tumor cell injections, mice were monitored on alternate days. The treatment groups consisted of mice that received nisin ZP at two different concentrations (400 and 800 mg/kg per day) and mice that received nisin AP at two different concentrations (400 and 800 mg/kg per day). There was also another group of mice that was given nisin ZP (800 mg/kg per day) over an extended period of time (months). Following completion of nisin administration, mice were euthanized by CO_2_ overdose and cervical dislocation, then tumors were harvested, rinsed in phosphate-buffered saline (PBS), imaged, and fixed overnight in 10% buffered formalin. A digital caliper was used to determine the tumor volume using the formula *a* x *a* x *b*/2, where *a* is the smaller dimension. In general, mice were euthanized if mice exhibited physiologic signs of stress from inability to eat or drink, weight loss, or when tumor volumes reach a range of 300–500mm^3^, and thus mice were generally euthanized at approximately 3 weeks for the short-term protocol. Mice were euthanized by a CO_2_ overdose.

### Statistical Analysis

In general, values were expressed as means ± SD. Intergroup differences were analyzed by the analysis of variance (ANOVA) and Tukey-Kramer HSD test. For the in vivo studies, independent t-tests with unequal variance were used.

## Results

### Nisin ZP and nisin AP induce significant HNSCC cell apoptosis dose-dependently and beyond that in low content nisin

**Fig 1:**
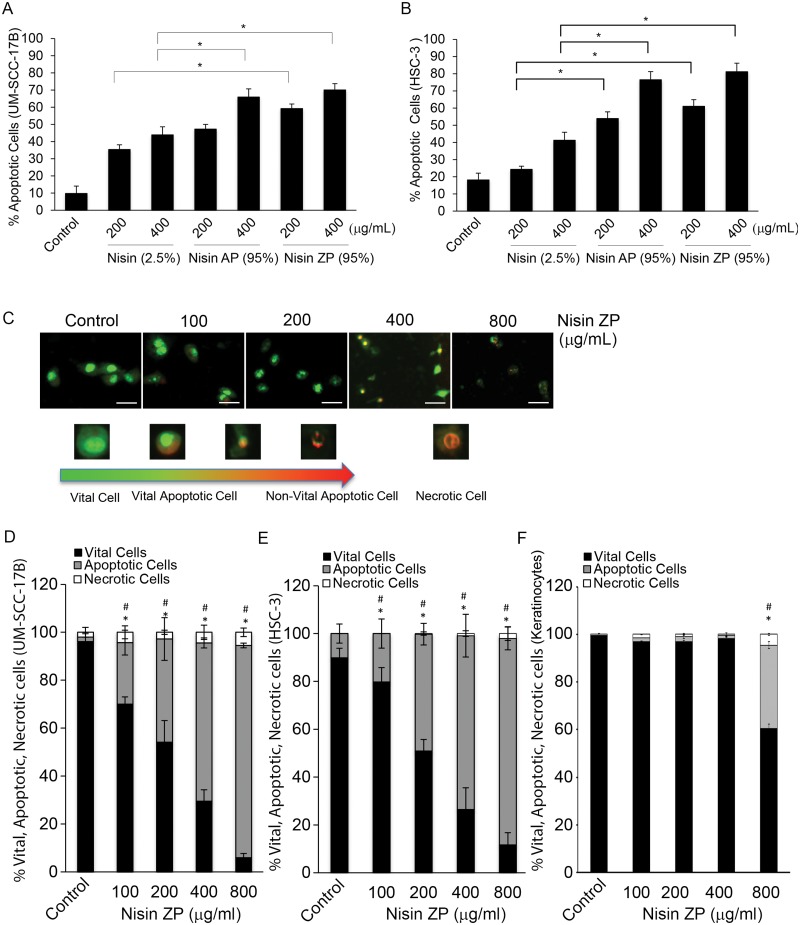
Nisin ZP and nisin AP induce significant HNSCC cell apoptosis dose-dependently and beyond that in low content nisin. *A and B*, Graphs showing changes in percentage of apoptotic HNSCC cells (HSC-3 and UM-SCC-17B) treated with control media or media containing 2.5% nisin, 95% nisin AP, or 95% nisin ZP for 24 h. Cells were then stained using an ethidium bromide and acridine orange (EB/AO) stain and counted. Comparisons between groups relative to controls were analyzed by ANOVA with the level of significance set at *p<0.05. *C*, Microscopic images showing morphologhy of HNSCC cells treated with control media or media containing nisin ZP (100 to 800 μg/mL) for 24 h then stained with EB/AO (Scale bars, 100 μm). *D-F*, Graphs showing changes in percentage of vital, apoptotic, and necrotic cells (UM-SCC-17B, HSC-3 and normal oral keratinocytes) treated with control media or media containing nisin ZP (400 or 800 μg/mL) for 24 h. Comparisons between groups relative to controls were analyzed by ANOVA with the level of significance set at *p<0.05 vital cells; # p<0.05 apoptotic cells.

Treatment with both nisin ZP (95%) and nisin AP (95%) induced significant increases in apoptosis in HNSCC cells (UM-SCC-17B and HSC-3) compared to treatment with low content nisin (2.5%) (Fig 1A and 1B). Apoptosis was measured using ethidium bromide and acridine orange (EB/AO) staining and microscopy. The effects of nisin ZP and AP on apoptosis were dose-dependent and increased as the concentrations of nisin ZP and AP increased from 200 to 400 μg/ml. Although the differences in apoptosis between nisin ZP and nisin AP were not significant, nisin ZP exhibited better solubility, and thus most experiments were performed with nisin ZP.

Furthermore, the effects of nisin ZP on HNSCC cell apoptosis were significant throughout a broad range of doses from 100, 200, 400 and 800 μg/mL (Fig 1C–1E) and in several HNSCC cell lines (UM-SCC-17B and HSC-3). Coordinately, cells treated with increasing doses of nisin ZP exhibited significantly decreasing numbers of vital cells and significantly increasing numbers of apoptotic cells, whereas the number of necrotic cells stayed relatively low and constant. In contrast, primary keratinocytes did not exhibit enhanced levels of apoptosis like that seen in HNSCC cell lines (Fig 1F).

### Nisin ZP induces apoptosis via calpain, caspase 8 and PARP cleavage

**Fig 2:**
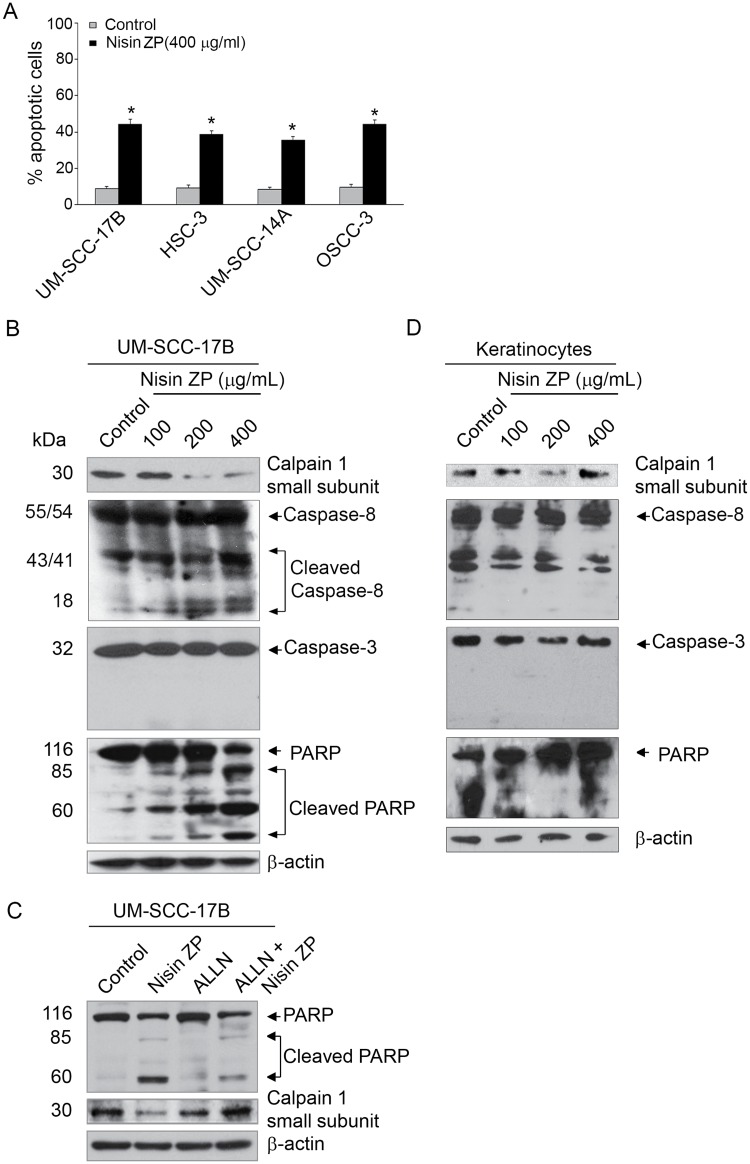
Nisin ZP induces calpain, caspase-8 and PARP activation in HNSCC cells dose-dependently. *A*, Graphs showing changes in percentage of apoptotic cells (UM-SCC-17B, HSC-3, UM-SCC-14A and OSCC-3) treated with control media or media containing nisin ZP (400 μg/mL) for 24 h. Cells were then stained with Annexin V and apoptosis was assessed by flow cytometry. Comparisons between groups relative to controls were analyzed by ANOVA with the level of significance set at *p<0.05. *B*, Immunoblots showing calpain 1, caspase-8, caspase-3, and PARP protein levels in HNSCC (UM-SCC-17B) and *C*, normal human oral keratinocytes cell lysates after treatment with nisin ZP for 18 h. *D*, Immunoblots showing PARP and calpain 1 activation in HNSCC cell lysates after treatment for 18 h with control media, media containing nisin ZP (400 μg/mL), media containing a calpain inhibitor (ALLN, 500 nM), or media containing both a calpain inhibitor (ALLN, 500 nM) and nisin (400 μg/mL). *D*, An immunoblot for ß-actin shows the loading controls.

To further examine the mechanism of nisin-mediated apoptosis, we employed additional apoptotic assays and surveyed known apoptotic signaling proteins. Four different HNSCC cell lines were examined for their nisin responsiveness. Nisin significantly increased apoptosis in all four HNSCC cells lines as assessed by annexin V staining and flow cytometry (Fig 2A).

Given our previous findings that nisin mediates calcium-dependent apoptosis, we explored the potential involvement of calpain, a known calcium dependent mediator of apoptosis [9]. Indeed, nisin treated HNSCC cells exhibited decreased expression levels of the calpain 1 small subunit, indicative of calpain 1 activation (Fig 2B). The decreasing expression of the calpain 1 small subunit mirrored the increasing doses of nisin ZP from 100 to 400 μg/mL, and thus was dose-dependent.

To further explore the mechanism of apoptosis induced by nisin ZP, caspase-8 and caspase-3 cleavage were examined in this context. Nisin induced caspase-8 activation in a dose-dependent manner (Fig 2B). However, treatment of HNSCC cells with nisin ZP induced caspase-3 independent apoptosis (Fig 2B). Western blotting for caspase-3 in nisin ZP treated cells revealed steady levels of caspase-3 expression and the absence of caspase-3 cleavage regardless of the nisin dose tested (Fig 2B).

Nisin ZP treated cells also exhibited poly (ADP-ribose) polymerase (PARP) cleavage, a hallmark of apoptosis (Fig 2B). PARP cleavage increased dose-dependently as nisin ZP doses increased. To determine whether nisin-mediated PARP cleavage was a calpain-dependent mechanism, a calpain inhibitor (ALLN) was examined in this context (Fig 2C). Treatment of HNSCC cells with both nisin ZP and a calpain inhibitor, effectively diminished PARP cleavage, compared to cells treated with nisin alone. Normal human oral keratinocytes did not exhibit activation of calpain, caspase-8, caspase-3 or PARP in response to treatment with nisin ZP (Fig 2D). Thus, nisin ZP induced apoptosis of HNSCC cells via activation/cleavage of calpain, caspase-8 and PARP, but independent of caspase-3 cleavage.

### Nisin ZP and nisin AP significantly reduce HNSCC cell proliferation time- and dose-dependently

**Fig 3:**
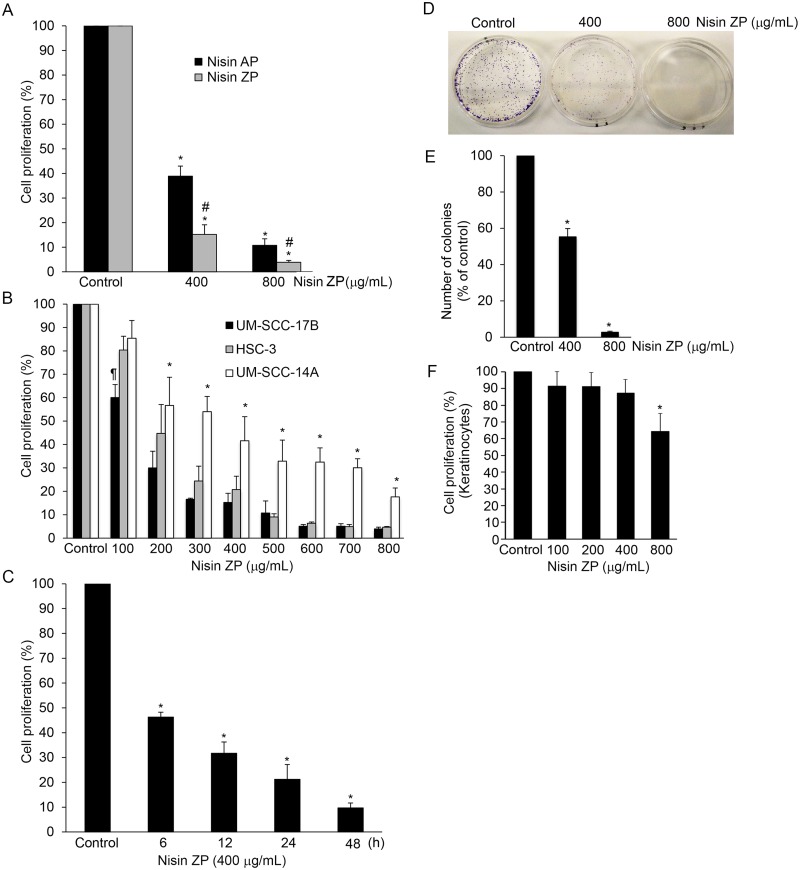
Nisin ZP and nisin AP significantly reduce HNSCC cell proliferation time- and dose-dependently and clonogenic capacity. *A*. Graphs showing changes in cell proliferation in UM-SCC-17B cells treated with control media or media containing nisin AP or ZP (400 or 800 μg/mL) for 48 h. Comparisons between groups relative to controls were analyzed by ANOVA with the level of significance set at *p<0.05. # Comparison between nisin AP and nisin ZP *p≤0.05. *B*. Graphs showing changes in cell proliferation in HNSCC cells (UM-SCC-17B, HSC-3 and UM-SCC-14A) treated with control media or media containing increasing doses of nisin ZP (100 to 800 μg/mL) for 48 h. Comparisons between groups relative to controls were analyzed by ANOVA with the level of significance set at *p<0.05. ¶Comparison between the 100 μg/ml group relative to the control for UM-SCC-17B cells *pμ0.05. *C*. Graphs showing changes in cell proliferation in UM-SCC-17B cells treated with control media or media containing nisin ZP (400 μg/mL) for 6, 12, 24 and 48 h. Comparisons between groups relative to controls were analyzed by ANOVA with the level of significance set at *p<0.05. *D*, The images are of UM-SCC-17B cells treated for 48h with control media or media containing nisin ZP (400 or 800 μg/mL) then cultured for 10 days, stained with crystal violet and imaged to evaluate clonogenic capacity. *E*, The graph shows the percentage of colonies present relative to controls for assays measuring clonogenic capacity. Comparisons between groups relative to controls were analyzed by ANOVA with the level of significance set at *p<0.05. *F*. Graphs showing changes in cell proliferation in normal human oral keratinocytes treated with control media or media containing nisin ZP (100, 200, 400 or 800 μg/mL) for 48 h. Comparisons between groups relative to controls were analyzed by ANOVA with the level of significance set at *p<0.05.

The effects of nisin ZP and AP on HNSCC cell proliferation were examined next, and we found that treatment with both nisin ZP and nisin AP significantly reduced HNSCC cell (UM-SCC-17B) proliferation (Fig 3A). The effects of nisin ZP and AP on HNSCC cell proliferation were dose-dependent, since proliferation levels decreased as the concentration of nisin ZP and AP increased from 400 to 800 μg/mL. In addition, the differences in HNSCC cell proliferation induced by nisin ZP versus nisin AP treatment were significant, such that the effects induced by nisin ZP were more pronounced. Thus, given the greater effects of nisin ZP in reducing proliferation and given the improved solubility of nisin ZP over nisin AP, nisin ZP was selected for further study. The effects of nisin ZP on HNSCC cell proliferation were significant throughout a broad range of doses from 100 to 800 μg/ml and in several HNSCC cell lines (Fig 3B). Also, the nisin ZP-mediated reduction in HNSCC cell proliferation was time-dependent, showing significant effects at 6, 12, 24, and 48 h (Fig 3C). The reduced cell proliferation likely reflects in part the cell cycle arrest mediated by nisin [9]; further evidenced by decreased phosphorylation of the cell cycle checkpoint marker, cdc2 (S1 Fig).

### Nisin ZP reduces HNSCC cell colony formation

We further explored the effects of nisin ZP on cell proliferation by measuring its long-term effects using colony formation assays. In agreement with the short-term proliferation assays, treatment with nisin ZP inhibited colony formation in HNSCC cells (Fig 3D and 3E). The inhibitory effects on colony formation were dose-dependent, since colony formation diminished significantly as the nisin ZP dose increased from 400 to 800 μg/mL. HNSCC cells treated with these two doses exhibited a 2 and 50-fold decrease in clonogenic capacity. Nisin did not inhibit proliferation in normal human oral keratinocytes like that in HNSCC cells (Fig 3F).

### Nisin ZP blocks orasphere formation

**Fig 4:**
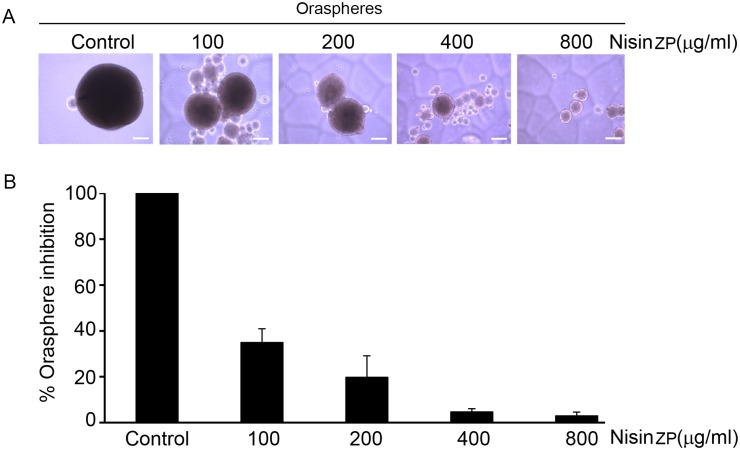
Nisin ZP inhibits orasphere formation in HNSCC cells. *A*, Phase contrast images and *B*, graph showing percentage of orasphere inhibition (total area) in HNSCC cells (UM-SCC-17B) cultured under suspension conditions and treated with control media or media containing nisin ZP (100 to 800 μg/ml) for 36 h. Comparisons between groups relative to controls were analyzed by ANOVA with the level of significance set at *p<0.05.

We further found that treatment with nisin ZP significantly blocked orasphere formation or anchorage independent growth (Fig 4A and 4B). Orasphere formation or anchorage independent growth is a property that contributes to tumorigenic potential. Nisin ZP’s effects on orasphere formation were dose-dependent, such that total orasphere area decreased steadily following treatment with nisin doses from 100, 200, 400 and 800 μg/mL.

### Nisin ZP induces endothelial cell apoptosis and inhibits angiogenic sprouting

**Fig 5:**
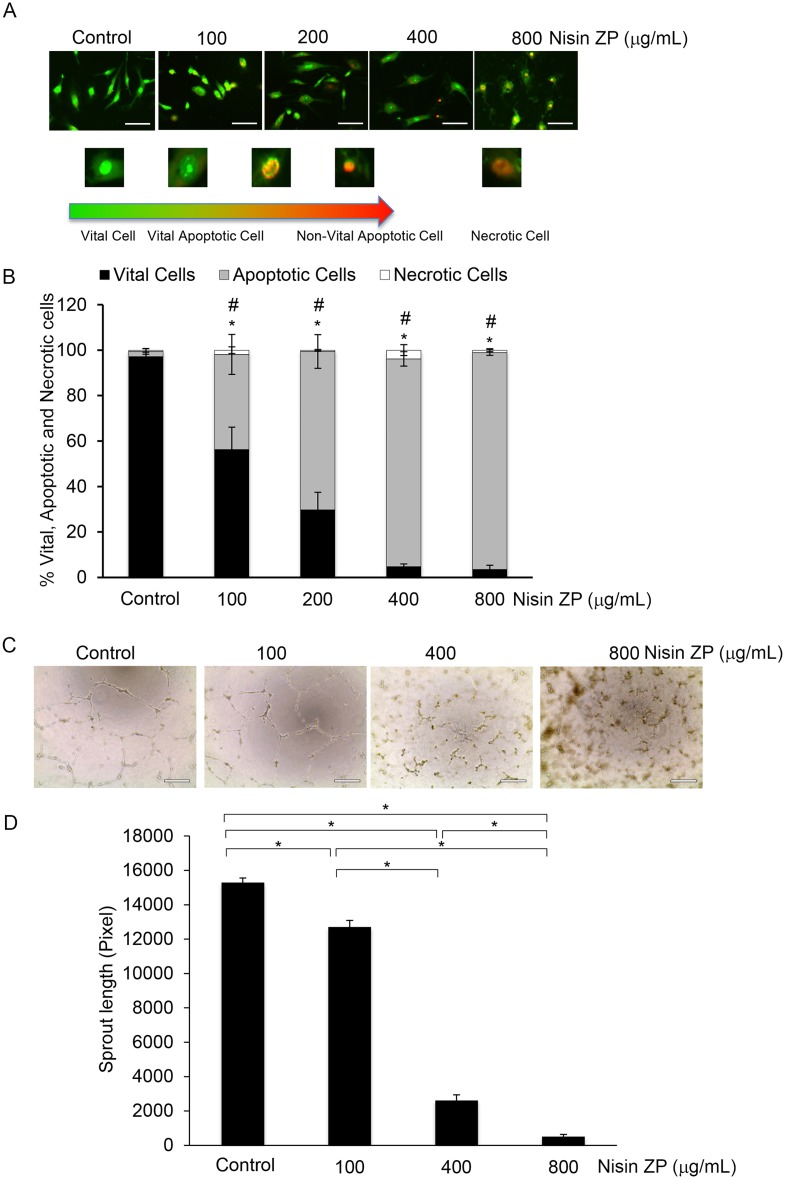
Nisin ZP induces endothelial cell apoptosis and inhibits angiogenic sprouting dose-dependently. *A*, Morphological observation of HUVEC cells treated with control media or media containing nisin ZP (100 to 800 μg/mL) for 24h. Cells were then stained with an ethidium bromide and acridine orange (EB/AO) stain, imaged, and counted (Scale bars, 100 μm). *B*, Graph showing percentage changes in vital, apoptotic, and necrotic HUVEC cells treated with control media or media containing nisin ZP (100 to 800 μg/mL) for 24 h. Comparisons between groups relative to controls were analyzed by ANOVA with the level of significance set at *p<0.05 vital cells; # p<0.05 apoptotic cells. *C*, Microscopic images of sprouting assays for cells (HUVEC) treated with control media or media containing nisin ZP (100 to 800 μg/mL) for 24h then imaged and counted (Scale bars, 100 μm). *D*, Graphs showing the total sprout length achieved by cells treated as indicated in C. Comparisons between groups relative to controls were analyzed by ANOVA with the level of significance set at *p<0.05.

We previously showed that treatment with low content nisin inhibited tumor growth and resulted in small pale tumors, suggesting that nisin affected the tumor blood supply. We also previously reported that nisin’s antitumor effects were mediated by CHAC1, a proapoptotic inducer in endothelial cells [9]. Thus, to determine whether nisin ZP affects angiogenesis, we evaluated the effects of nisin ZP on endothelial cell apoptosis and sprouting. Human umbilical vein endothelial cells (HUVECs) treated with different concentrations of nisin ZP (100, 200, 400 and 800 μg/mL) exhibited significantly increasing levels of apoptosis when compared to cells treated with media control (Fig 5A and 5B). Consistently, the vital cell fraction decreased coordinately with increasing doses of nisin, whereas the necrotic cell fraction remained small and constant throughout the treatment with different nisin doses. HUVECs treated with different concentrations of nisin ZP (100, 400 and 800 μg/mL) also exhibited significantly decreased sprouting when compared to cells treated with the media control (Fig 5C and 5D). Thus, nisin ZP induces significant endothelial cell apoptosis and inhibits angiogenic sprouting dose-dependently. Nisin may thereby promote further tumor reduction due to its anti-apoptotic and anti-proliferative effects on both tumor cells and endothelial cells.

### Nisin AP and ZP reduces HNSCC tumor burden in vivo

**Fig 6:**
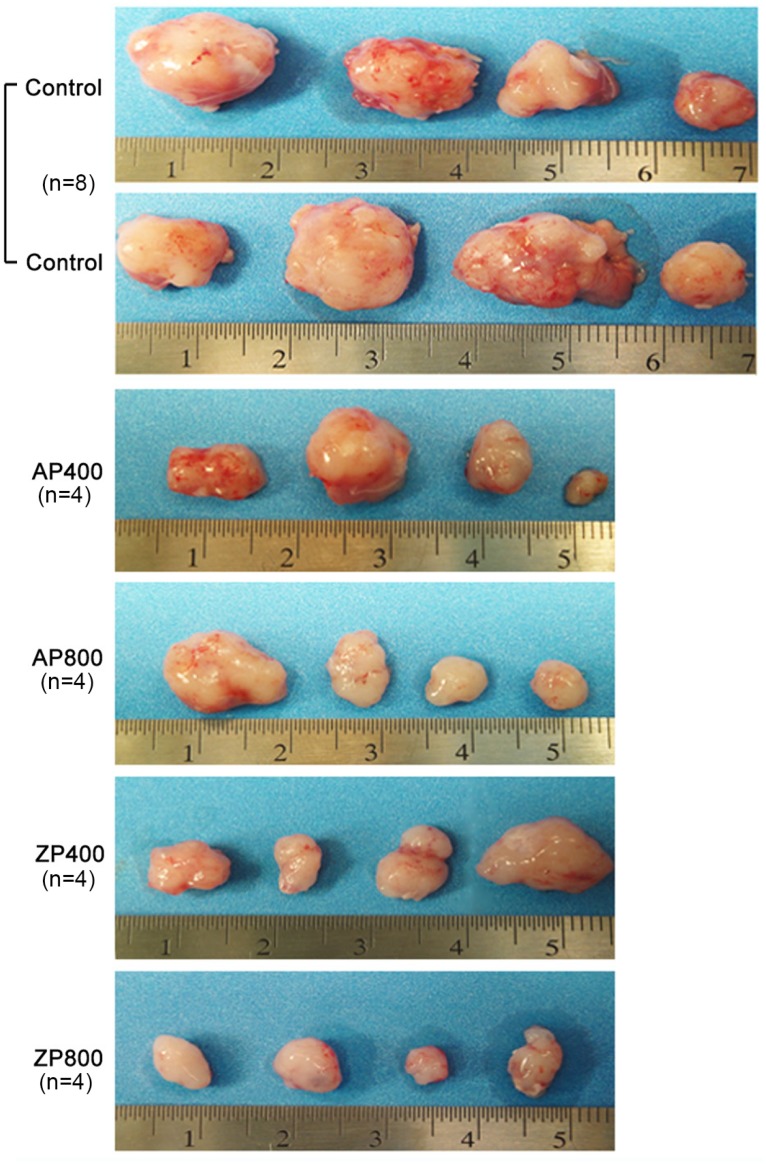
Nisin ZP reduces HNSCC tumor burden in mice. Images show the dissected tumors obtained from mice injected with UM-SCC-17B cells then treated with either water (control) or nisin AP or nisin ZP (400 or 800 mg/kg body weight/day) for 3 weeks.

To further study the effect of nisin ZP on HNSCC tumorigenesis, we used a well-characterized floor-of-mouth oral cancer xenograft mouse model (Fig 6, Table 1). After 3 weeks of treatment, mice receiving nisin ZP and nisin AP exhibited significantly reduced tumor volumes compared to controls (Fig 6, Table 1). All concentrations of nisin tested significantly reduced mean tumor volumes (13.5, 88.5, 59.2, and 66.75 mm^3^ for nisin ZP 800 mg/kg, nisin ZP 400 mg/kg, nisin AP 800 mg/kg, and nisin AP 400 mg/kg, respectively) compared to controls (232.8 mm^3^). In general, control treated mice exhibit physiologic signs of stress from inability to eat or drink when tumor volumes reach a range of 300–500mm^3^, and thus mice were generally euthanized at approximately 3 weeks for the short-term protocol.

**Table 1:** Tumor volumes for mice injected with UM-SCC-17B cells then treated with water (control) or nisin (400 or 800 mg/kg body weight/day), using a short term (3 weeks) or long term treatment approach (9 weeks).

Short Term Treatment						Long Term Treatment	
Animal number	Control	AP400	AP800	ZP400	ZP800	Animal Number	ZP800
	(mm3)	(mm3)	(mm3)	(mm3)	(mm3)		(mm3)
1	374	57	191	39	12	1	356
2	202	179	24	13	25	2	395
3	75	26	9	68	4	3	91.9
4	50	5	13	234	13	4	126
5	204						
6	584						
7	299						
8	75						
**Mean volume**	**232.8**	**66.75** *	**59.2** *	**88.5** *	**13.5** *		**242** *

Statistical analysis: Independent t-test with unequal variances

*p<0.05.

**Fig 7:**
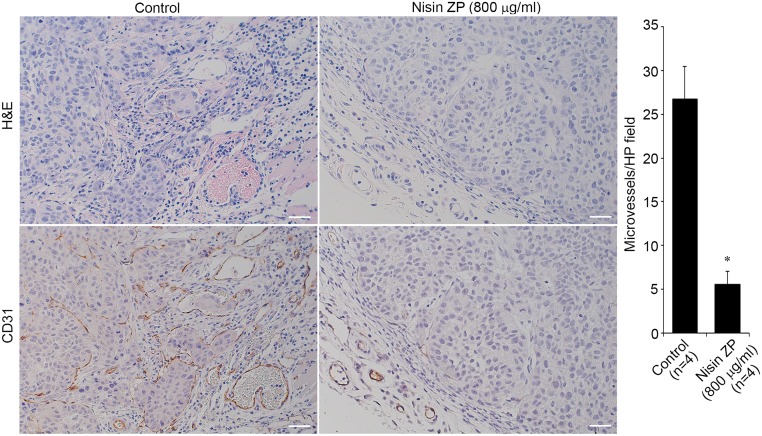
Nisin ZP reduces tumor microvessel density. *A*, Representative images of histological sections stained with H&E (top), and histological sections immunostained for CD31 (bottom) to identify blood vessels. *B*, Graph showing the results of microvessel quantification from five high power fields per tumor (*p<0.05).

**Fig 8:**
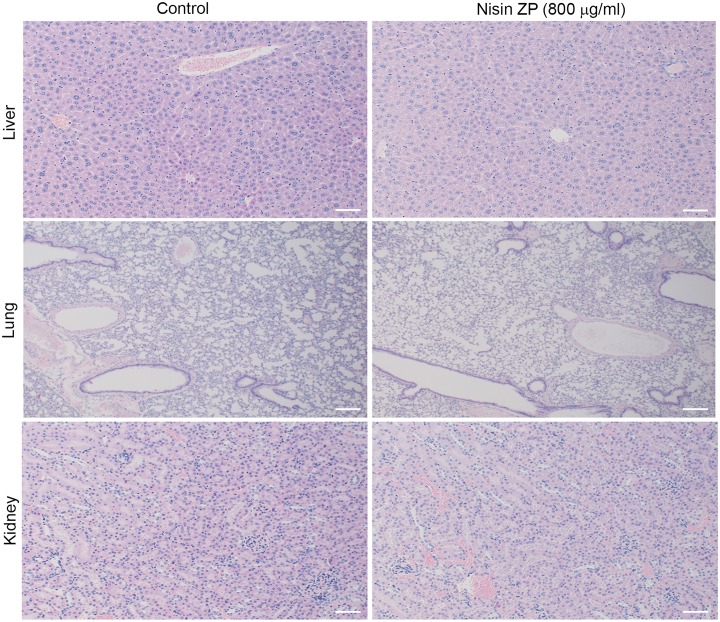
Nisin ZP does not elicit histological signs of toxicity in the liver, lung and kidney of mice. Images of H&E-stained histological sections of liver, lung and kidney from mice injected with UM-SCC-17B cells then treated with either water (control) or nisin ZP (800 mg/kg body weight/day) for 3 weeks.

In contrast, mice in the long-term treatment group, which were given 800 mg/kg nisin ZP, survived extended periods of time without compromising eating or drinking. At 9 weeks, tumor volumes for this group of mice were as follows: mouse 1 was 356 mm^3^, mouse 2 was 395 mm^3^, mouse 3 was 91.9 mm^3^, and mouse 4 was 126 mm^3^ (Table 1). The survival times for these mice were as follows: mouse 1 survived 9 weeks, mouse 2 survived 9 weeks, mouse 3 survived 12 weeks, mouse 4 survived 16 weeks. Additionally, tumor volume for mouse 3 was 367 mm^3^ (12 weeks) and mouse 4 was 2745 mm^3^ (16 weeks) at euthanasia. Despite the large tumor volume in mouse 4, eating and drinking were not compromised until the latter time points. For this long treatment group of mice the mean tumor volume was 242 mm^3^ at 9 weeks (Table 1). Importantly then, the mean tumor volume for control treated mice at 3 weeks was 232.8 mm^3^, whereas the equivalent mean tumor volume in the nisin treated mice (242 mm^3^) was reached at 9 weeks. Thus nisin treatment extended the time required for mice to reach comparable tumor volumes. Furthermore, nisin ZP treatment reduced intratumoral microvessel density (Fig 7). There were no histological differences in the liver, lung, and kidney organs of nisin treated mice compared to the control treated mice (Fig 8). Specifically, there was no histological evidence of inflammation, fibrosis or necrosis in these organs following nisin treatment even at the highest tested dose of 800 μg/ml.

## Discussion

The current data on nisin ZP extend the baseline information for nisin and support the concept that nisin ZP may be a useful therapeutic for HNSCC, since nisin ZP promotes HNSCC cell apoptosis, suppresses HNSCC cell proliferation, and blocks angiogenic processes, orasphere formation and tumorigenesis in vivo. Translation of nisin ZP from the mouse setting to the human setting requires a dose conversion. Using the standard national cancer institute/national institute of health (NCI/NIH) dosage conversion factor of 12 for mouse to man an 800 mg/kg body weight/day of nisin ZP given to mice would translate to 66.7 mg/kg for humans [33]. FDA has accepted the highest dose tested in the Frazer et al., study (3,330,000 units of nisin per kg of diet, equivalent to 83.25 mg per kg of diet) as the no-observed-effect-level (NOEL) and affirmed the generally regarded as safe (GRAS) status of nisin derived from *Lactococcus lactis* [12,34]. Typically, nisin is added to foods in the range of 0.25 to 37.5 mg/kg [35]. Also, a manufacturer of a liquid pure nisin A (>2.5% concentration) recommends adding 7.5–100 ml/L in beverages, including fruit juices and alcoholic beverages [36]. Acute toxicity or the LD_50_ in male and female rates was reported to be 9.2 g/Kg and 8.81 g/Kg and 8.81 g/Kg, respectively [36]. Thus, a theoretical therapeutic dose for nisin (66.7 mg/kg) in humans would be approximately twice that already present in some foods (37.5/mg/kg). In addition, this dose does not approximate the reported toxic doses in rodents and is within the NOEL designation by the FDA.

Bacteriocins, are antimicrobial peptides that have been examined to a limited extent for their antineoplastic potential. Purified bacteriocins, including pyocin, colicin, pediocin, and microcin have shown inhibitory properties against neoplastic cell lines and in xenograft mouse models [37–47]. Nisin has already been used effectively in vivo as a therapeutic for infections. In humans, nisin was an effective alternative to antibiotics for the treatment of staphylococcal mastitis during lactation in women and it was used as a sanitizer against the mastitis pathogens *Staphylococcus* and *Streptococcus* species in lactating cows [48–50]. Our recent work also supports nisin as a potential therapeutic for HNSCC. Thus, given nisin’s known safety and use for over 40 years as a food preservative, its antitumor effects in vitro and in vivo, its use in humans as a therapeutic to treat infections, nisin ZP stands poised for development as a cancer therapeutic for HNSCC.

Although, a caspase cascade marked by activation and cleavage of caspase-3 is a hallmark of apoptosis, there are reports of caspase-3 independent apoptosis [51]. For example, p53-mediated cell cycle arrest and apoptosis is mediated through a caspase-3-independent, but caspase-9-dependent pathway in MCF-7 human breast cancer cells and leukemia cells [52,53]. Other mediators of apoptosis, such as calpain have been implicated in both caspase-3 dependent and independent apoptosis [54–60]. Calpain activation via a Ca2^+^ flux plays an essential role in eliciting an apoptosis-inducing factor (AIF)-mediated caspase-independent apoptosis and necroptosis in HeLa cells [61,62]. Additionally, CD44 ligation induces caspase-independent cell death via a novel calpain/AIF pathway in human erythroleukemia cells and U87MG human gliomas [63,64]. Thus, we explored the role of calpain in this study. Our data support the concept that nisin ZP induces a calpain-dependent mechanism of apoptosis that does not depend on caspase-3 cleavage (Fig 2B). In summary, nisin ZP, via induction of calpain-dependent apoptosis in HNSCC cells, induction of apoptosis in endothelial cells, and inhibition of HNSCC cell proliferation, inhibits tumorigenesis that exhibits reduced intratumoral microvessel density. Nisin ZP’s in vivo antitumor effects and known safety as a food preservative, position nisin ZP for future development as a therapeutic for HNSCC.

## Supporting Information

S1 Fig Nisin treatment decreases cdc2 phosphorylation in HNSCC cells. Western blots showing that nisin treatment (80 μg/ml nisin 2.5% purity; 24 h) decreases phosphorylation of the cell cycle checkpoint marker, cdc2, in HNSCC cells (UM-SCC-17B) compared to control/media treatment. A western blot for cdc2 shows that total protein levels for cdc2 do not change with nisin treatment. ß-actin served as a loading control.
(EPS)
